# Biodegradable Polymer DES (Ultimaster) vs. Magnesium Bioresorbable Scaffold (BRS Magmaris) in Diabetic Population with NSTE-ACS: A One-Year Clinical Outcome of Two Sirolimus-Eluting Stents

**DOI:** 10.1155/2021/8636050

**Published:** 2021-11-23

**Authors:** Piotr Rola, Adrian Włodarczak, Mateusz Barycki, Marek Szudrowicz, Magdalena Łanocha, Jan Jakub Kulczycki, Karol Turkiewicz, Katarzyna Woźnica, Maciej Lesiak, Adrian Doroszko

**Affiliations:** ^1^Department of Cardiology, The Copper Health Centre (MCZ), 59-300 Lubin, Poland; ^2^Department of Cardiology, Provincial Specialized Hospital in Legnica, 59-220 Legnica, Poland; ^3^Adalbert's Hospital, 61-144 Poznan, Poland; ^4^Faculty of Mathematics and Information Science, Warsaw University of Technology, 00-662 Warsaw, Poland; ^5^1st Department of Cardiology, Poznan University of Medical Sciences, 61-491 Poznan, Poland; ^6^Department of Internal Medicine, Hypertension and Clinical Oncology, Wroclaw Medical University, 50-556 Wroclaw, Poland

## Abstract

**Background:**

Cardiovascular disease (CVD) with significant involvement of coronary artery disease (CAD) remains a major cause of death and disability among the diabetic population. Although percutaneous coronary intervention (PCI) continues to evolve, type 2 diabetes mellitus (T2DM) is a well-established marker of poor clinical prognosis after PCI, which is mainly attributed to the rapid progression of atherosclerosis requiring recurrent revascularizations. Hence, the use of bioresorbable materials could provide some solution to this problem. *Material and Methods.* The study was divided into two arms. For the first one, we qualified 169 patients with NSTE-ACS treated with PCI who received the drug-eluting stent (DES) coated with a biodegradable polymer Ultimaster (Terumo, Tokyo, Japan). The second arm was composed of 193 patients with ACS who underwent PCI with a magnesium bioresorbable scaffold Magmaris (Biotronik, Berlin, Germany). Both arms were divided into two subsequent groups: the T2DM (59 and 72) and the non-DM (110 and 121, respectively). The primary outcomes were cardiovascular death, myocardial infarction, and in-stent thrombosis. The main secondary outcomes included target lesion failure (TLF) and were recorded at a 1-year-follow-up.

**Results:**

There were no significant differences between the diabetic and nondiabetic populations in the primary endpoints or main secondary endpoints (TLF, scaffold restenosis, death from any reason, and other cardiovascular events) either in the Ultimaster or Magmaris group. At a 1-year follow-up, the primary endpoint in the DM t.2 population was recorded in 2.7% Ultimaster vs. 5.1% Magmaris, respectively.

**Conclusion:**

Both, Ultimaster and Magmaris revealed relative safety and efficiency at a one-year follow-up in the diabetic population in ACS settings. The observed rates of TLF were low, which combined with a lack of in-stent thrombosis suggests that both investigated devices might be an interesting therapeutic option for diabetics with ACS. Nevertheless, further large randomized clinical trials are needed to confirm fully our results.

## 1. Introduction

Among patients with acute coronary syndrome, diabetes mellitus in particular is a marker of poor clinical prognosis. Diabetics tend to have rapid progression of atherosclerosis, leading to an increased rate of multivessel disease, which commonly requires recurrent revascularization. According to the current European Society of Cardiology (ESC) guidelines on myocardial revascularization [1], coronary artery bypass grafting (CABG) is preferred over percutaneous coronary intervention in diabetic patients. This recommendation is strictly related to a higher rate of short- and long-term adverse cardiovascular outcomes demonstrated after PCI. However, due to the aging and numerous comorbidities, PCI often remains the only available revascularization option. Many factors are postulated to play a role in the pathophysiological background of unfavorable results. Chronic vascular inflammation, endothelial dysfunction with increased oxidative stress, and increased platelet activation are cardiovascular responses to hyperglycemia [2]. In addition, these chronic inflammatory responses are often exacerbated by the drug-eluting stent [3] which can lead to delayed endothelialization of stent and subsequently impaired vascular healing process. To overcome these limitations, the bioresorbable materials have been widely used to develop new generations of scaffolds. These devices focus on suppressing the persistent inflammatory stimulus of the vascular wall by the stent surface.

Recently, the new generation of sirolimus-eluting bioresorbable polymer DES Ultimaster (Terumo, Tokyo, Japan) has demonstrated a favorable 1-year safety and efficacy profile with concomitant rapid vascular wall healing and a high degree of strut coverage [4]. A thin, biodegradable gradient coating is a novel feature of the scaffold design. Thus, the bioresorbable DES technology refers not only to the polymer but also the entire stent platform. Bioresorbable vascular scaffolds (BRS) constitute a novel vessel-supporting technology that enables the vessel restoration without permanent presence of foreign material in the vessel wall. The initial enthusiasm for the first generation of BRS Absorb (Abbott, Chicago, United States) subsided following publication of the long-term results [5]. However, the second generation of magnesium BRS Magmaris (Biotronik, Berlin, Germany) has recently entered the market and has shown promising short-term outcomes [6].

The aim of this study is to investigate the performance of sirolimus-releasing bioresorbable polymer stents (Ultimaster) compared to bioresorbable magnesium scaffold (Magmaris) and to evaluate the theoretical advantages of this new technology in high-risk population patients with diabetes mellitus in the setting of ACS.

## 2. Materials and Methods

**Figure 1:**
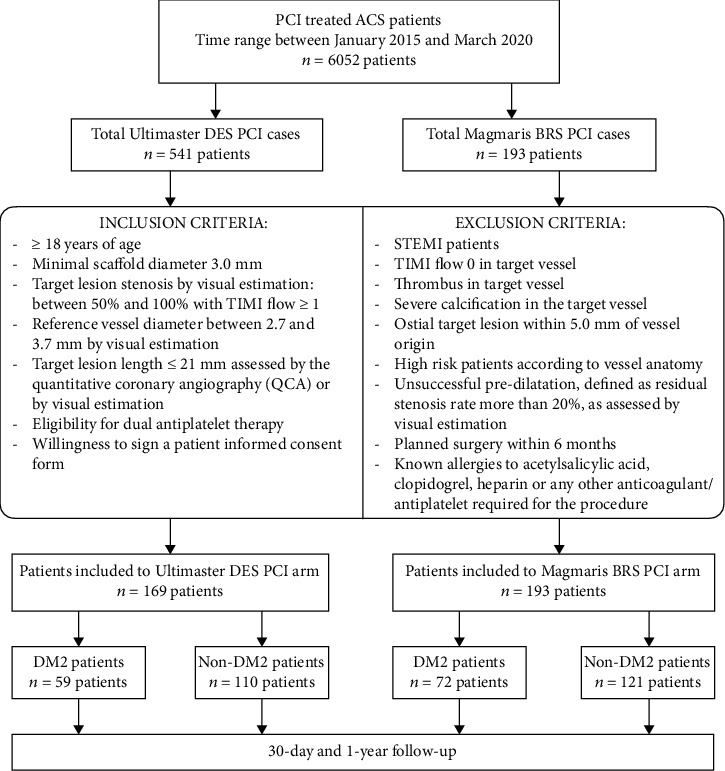
Study inclusion and exclusion criteria.

Patients with acute coronary syndrome–NSTE-ACS (with exclusion of the STEMI cases) and clinical indication for percutaneous coronary intervention (PCI) were enrolled in this retrospective, observational, study. This study consisted of two major arms (Figure 1). The first arm included 193 patients who received a bioresorbable magnesium scaffold—Magmaris. The second arm was composed of 169 patients who were implanted with a scaffold covered with a biodegradable polymer—Ultimaster. The decision to implant Magmaris BRS was based on operator dissertation in accordance with the inclusion and exclusion criteria (Figure 1), which were closely followed the manufacturer's recommendations [7]. Patients in the second arm were selected among all ACS-Ultimaster cases (541) from our cardiac departments between January 2015 and March 2020. The criteria for inclusion in the registry were the same as for the Magmaris group. In addition, scaffolds in the Ultimaster group—in parallel to the Magmaris group—had to meet the additional size-related criteria (diameter 3.0 mm or 3.5 mm).

### 2.1. Devices

Magmaris is a novel metallic (magnesium) sirolimus-eluting scaffold coated with a biodegradable polymer (BIOlute) poly-L-lactide (PLLA). Currently, available scaffold sizes are 3.0 and 3.5 mm in diameter and 15, 20, and 25 mm in lengths. Ultimaster is a cobalt-chromium sirolimus-eluting stent covert abluminal with a biodegradable poly-(D, L-lactide-co-caprolactone) copolymer (PDLLA-PCL).

### 2.2. Coronary Stenting Procedure

All patients receive a periprocedural medication regimen according to the routine practice in accordance with current revascularization guidelines [8]. Initially, mandatory aggressive (balloon-artery ratio 1 : 1 size according to angiographic assessment) and successful (without significant more than 20% of diameter-residual stenosis) lesion preparation was performed. In the next step, after successful stent delivery and implantation, obligatory high-pressure (at least 15 atm.) postdilation was performed with a NC balloon, which has a size at least equal to the size of the scaffold.

### 2.3. Endpoints and Definitions

The primary outcome included death from cardiac causes, myocardial infarction, and stent thrombosis. The main secondary outcome was a target-lesion failure (TLF) composed of cardiac death, target vessel myocardial infarction (TV-MI), or target lesion revascularization (TLR). Also, other secondary outcomes (scaffold restenosis, death from any reason, and all revascularization procedures as well as myocardial infarction [9]) were recorded.

Diabetes (type 1 or type 2) was defined as a previously diagnosed DM treated with pharmacologic or nonpharmacologic, and a new-onset DM was defined according to the American Diabetes Association [10].

### 2.4. Statistical Analysis

The analyses were performed using the R language [11]. Continuous variables were characterized with their mean and standard deviation, while frequencies were used for categorical variables. Patients were compared between groups using the nonparametric two-sample Mann–Whitney's test for continuous variables and Fisher's exact test for categorical variables. Bonferroni correction was applied to adjust for multiple comparisons, *p* values ≤ 0.05 were accepted as a threshold for statistical significance.

## 3. Results

The Magmaris group consisted of 72 diabetics and 121 nondiabetic cases. In the diabetic group, the majority of patients received oral antidiabetic treatment rather than insulin (58 (80.5%) vs. 14 (19.5%)). The diabetic group had a significantly higher prevalence of hypertension (95.8% vs. 84.2%, respectively, *p* = 0.018) and a past history of PCI (50% vs. 34.7%, respectively, *p* = 0.048) as well as was characterized by a significantly lower left ventricular ejection fraction (57.7% vs. 59.4%, respectively, *p* = 0.050) In contrast, a nondiabetic Magmaris group had more severe initial lipid disorders—total cholesterol (4.8 ± 1.3 vs. 4.3 ± 1.3 mM, respectively, *p* = 0.008) and LDL (2.8 ± 1.2 vs. 2.1 ± 0.9 mM, respectively, *p* < 0.001).

To the Ultimaster arm, we recruited a total of 59 diabetic subjects and 110 patients to the control group. Among the diabetic participants, the minority was treated with insulin (23.7%). There were no statistically significant differences in comorbidities between the diabetic and nondiabetic Ultimaster populations. The nondiabetics had higher serum level of total cholesterol (5.2 ± 1.4 vs. 4.5 ± 1.3 mM, respectively, *p* = 0.002) and LDL (2.5 ± 1.2 vs. 3.2 ± 2.1 mM, respectively, *p* < 0.002).  Table 1 summarizes the baseline clinical characteristics of the two arms.

The characteristics of the PCI procedures performed in both study arms were heterogeneous. The only statistically significant differences were found in the Ultimaster arm and related to the radiation dose used during the PCI procedure, which was higher in the diabetic group (1396.56 ± 802.95 vs. 1162.52 ± 728.34, respectively, *p* = 0.029). All procedural characteristics are shown in Table 2.

All clinical outcomes data are summarized in Tables 3 and 4. There were no statistically significant differences in clinical outcomes between the diabetic and control populations in either study arms (Magmaris and Ultimaster). We did not find any significant differences between the two diabetic study populations (Magmaris vs. Ultimaster). The only exception was a higher number of all types—revascularization at 30-day follow-up in the diabetic Ultimaster group, compared to the diabetic Magmaris group (5 vs. 0, respectively, *p* = 0.016). Noteworthy, the rates of the primary outcome were higher in the diabetic population in the Ultimaster group (3.4% vs. 0%, respectively, *p* = 0.121) at short follow-up (30 days). A similar trend was observed at long-term follow-up (1 year) for principal secondary outcome in the Magmaris arm (4.1% vs. 0%, respectively, *p* = 0.051).

## 4. Discussion

Despite worldwide public health interventions taken to stop the global growth of diabetes prevalence, it is inexorably increasing. A disproportionate burden of the increase in type 2 diabetes affects the middle-to-high-income countries, particularly Western Europe and the Pacific Ocean island nations [12]. Cardiovascular disease (CVD) including coronary artery disease (CAD) as a major contributor, remains a leading cause of death and disability among the diabetic population. Although percutaneous coronary intervention (PCI) continues to evolve, the data from randomized trials demonstrate the superiority of coronary artery bypass grafting (CABG) over percutaneous coronary intervention in the diabetic population [13]. The reasons for this are multifactorial and not fully understood. Some data link this to a chronic local inflammatory response in response to the presence of a foreign body in the vessel wall, leading to neointimal hyperplasia and increased platelet activation and adhesion [14]. Therefore, the use of bioresorbable material design to limit immune-adverse reactions is believed to be a new revolution in the field of coronary interventions. In current practice, two development paths for bioresorbable materials have been proposed.

The first, also referred to as third-generation DES, involves abluminal coating of a thin metallic backbone with a bioresorbable polymer that degrades uniformly to release the antimitotic drug sirolimus. An example of this technology is the Ultimaster.

The second concept pursues complete biosorption of the scaffold. In this scenario, BRS provides short-term performance equivalent to existing drug-eluting stents (DES); however, it avoids permanent caging of the vessel. After the widespread use of first-generation Absorb (Abbott) was discontinued, the second generation of BRS (Magmaris) with a metallic backbone (magnesium) sirolimus-eluting BRS containing an active bioabsorbable coating BIOlute poly-L-lactide (PLLA) entered the market and is currently available for commercial use.

Data on the performance of the Ultimaster in the all-comers population are encouraging and demonstrated low late lumen loss, resulting in low rates of in-stent thrombosis, restenosis, and TLR [15–17]. Clinical outcomes in the long-term follow-up were comparable to those obtained with the Xience scaffolds [18]. The long-term safety of Ultimaster was confirmed by the low rate of late in-stent thrombosis. These favorable antithrombotic properties of the scaffold have been demonstrated in the *in vitro* models [19] and are associated with an accelerated tissue coverage and scaffold apposition [3, 20] leading to improved vessel healing. Noteworthy, the presence of the “class effect” for all bioresorbable polymer stents is very likely [21].

It is well known that diabetes mellitus and ongoing ACS are independent risk factors for poor clinical outcomes after PCI. Although there is a lack of convincing data for Ultimaster, few studies conducted so far seem to confirm this paradigm [22, 23] mainly due to an increased rate of TLF. However, the data from our studies do not confirm this observation. There were no statistical differences between the diabetic and control groups in primary clinical outcomes and TLF. Moreover, the rate of TLF in diabetics was significantly lower than in the study of Beneduce et al. [23] (3.3% vs. 8%). A similar trend is observed when we consider substudies in the ACS group [24]. This could be due to the fact that only patients implanted according to the accordance “4P technique“(patient selection, proper sizing, predilatation, and postdilatation strategy) were analyzed. It has been shown that the negative effects of diabetes on patients treated with BRS-ABSORB implantation can be minimized [25, 26].

On the other hand, our favorable results may be related to the detailed lesion selection that we adopted from inclusion criteria of the Magmaris Registry. We avoided high-risk patients with heavy calcification, the STEMI patients with present thrombus, or low-size of the treated vessel. However, the latter factor has been proven to have no effect on clinical outcome after Ultimaster implantation [22, 23]. Therefore, concerning the results of the LEADERS trial [27], it seems to be no “class effect” of DES with abluminal biodegradable coating.

Data regarding the performance of Magmaris in the diabetic population are strictly limited [28], yet encouraging. In the contrast, the data on implantation of Magmaris in ACS conditions are more comprehensive and reliable. Several observational registries confirmed favorable short-term and long-term outcomes [6, 29] Furthermore, recently published data from the largest all-comers Magmaris registry [30] which included 2054 subjects showed that the one-year TLF rate was 4.3% with only one subacute in-stent thrombosis event. The results obtained are far more favorable than the first generation of BRS and comparable to the newest DES. There is only one study comparing Magmaris to third generation of DES (Orsiro) [31]. The study population consisted mainly of the patient with stable CAD. The authors observed Magmaris and Orsiro unadjusted TLF rates at levels 6.0 and 6.4% with no significant difference between the groups.

To the best of our knowledge, this is the first in human study designed to compare the efficacy and safety of fully bioresorbable magnesium scaffold (Magmaris) with a third generation of metallic DES with bioresorbable polymer, in DM t.2 population in ACS settings. We found no differences between the two scaffolds in the diabetic subpopulation. As reported in our study, the 1-year TLF in DM subpopulation for both devices (4.1% vs. 3.3%) is comparable [30] and even better [23, 31] than in the previously mentioned studies. Diabetes, especially when treated with insulin, is a well-established risk factor of scaffold thrombosis, particularly in the first generation of BRS (Absorb) [32]. Our data contradict such an association. We did not observe the in-stent thrombosis in any of the tested devices. Noteworthy, both used scaffolds released the same antimitotic drug (sirolimus), and therefore, the results are not differentiated by this factor.

### 4.1. Limitations

This was a nonrandomized study with retrospective data collected in the relatively short observation period (1-year follow-up). The study population was not very large and underpowered for reliable assessment of events, especially in the diabetic subpopulation. Also, the rate of intravascular guidance PCI was comparatively low.

## 5. Conclusions

In our study both biodegradable polymer DES (Ultimaster) and Magnesium bioresorbable scaffold (Magmaris) revealed relative safety and efficiency features at a one-year follow-up in the diabetic population in ACS settings. The observed rates of TLF were low, which combined with a lack of in-stent thrombosis suggests that both investigated devices might be an interesting therapeutic option for diabetics with ACS. Nevertheless, further large randomized clinical trials are needed in order to confirm fully our results.

## Figures and Tables

**Table 1:** Baseline clinical characteristics of both study arms.

	Magmaris group			Ultimaster group		
	Diabetes (*N* = 72)	Nondiabetes (*N* = 121)	*p* value	Diabetes (*N* = 59)	Non diabetes (*N* = 110)	*p* value
Age (years)	65.3 ± 7.9	63.2 ± 9.5	*p* = 0.127	66.1 ± 9.1	64.8 ± 9.5	*p* = 0.363
NSTEMI	58 (80.5%)	92 (76.0%)	*p* = 0.592	32 (54.2%)	56 (50.1%)	*p* = 0.628
Unstable angina	14 (19.5%)	16 (13.2%)	*p* = 0.305	27 (45.8%)	54(49.9%)	*p* = 0.628
Oral anti-diabetic treatment	58 (80.5%)	NA	—	45 (76.3%)	NA	—
Insulin	14 (19.5%)	NA	—	14 (23.7%)	NA	—
Hypertension	69 (95.8%)	102 (84.2%)	*p* = 0.018	58 (98.3%)	100 (90.1%)	*p* = 0.099
Hyperlipidemia	58 (80.5%)	94 (77.0%)	*p* = 0.718	47 (79.6%)	83 (75.5%)	*p* = 0.682
Atrial fibrillation	2 (2.7%)	7 (5.7%)	*p* = 0.488	11 (18.6%)	13 (11.8%)	*p* = 0.099
Post PCI status	36 (50%)	42 (34.7%)	*p* = 0.048	27 (46.5%)	34 (30.9%)	*p* = 0.061
Primary diagnosis of MI	28 (38.8%)	31 (25.6%)	*p* = 0.075	26 (44.1%)	34 (30.9%)	*p* = 0.063
LVEF	57.7% ± 10.7	59.4% ± 16.0	*p* = 0.050	54.1% ± 13.4	57.9% ± 27.2	*p* = 0.271
Total cholesterol (mmol/L)	4.3 ± 1.3	4.8 ± 1.3	*p* = 0.008	4.5 ± 1.3	5.2 ± 1.4	*p* = 0.002
LDL (mmol/L)	2.1 ± 0.9	2.8 ± 1.2	*p* < 0.001	2.5 ± 1.2	3.2 ± 2.1	*p* = 0.002
Triglycerides (mmol/L)	1.9 ± 1.1	1.8 ± 2.1	*p* = 0.213	1.6 ± 0.8	1.6 ± 0.8	*p* = 0.889
Creatine (*μ*mol/L)	82.3 ± 21.5	85.1 ± 22.5	*p* = 0.431	85.5 ± 22.4	81.6 ± 21.6	*p* = 0.378

Abbreviations: NSTEMI: no ST-elevation myocardial infarction; PCI: percutaneous coronary intervention; MI: myocardial Infarction; LVEF: left ventricle ejection fraction.

**Table 2:** Procedural characteristics of both study arms.

	Magmaris group			Ultimaster group		
Procedural characteristic	DM (*N* = 72)	Non-DM (*N* = 121)	*p* value	DM (*N* = 59)	Non-DM (*N* = 110)	*p* value
Treated vessel: LAD	31 (43%)	49 (40.5%)	*p* > 0.999	21 (35.5%)	44 (40%)	*p* > 0.999
LCX	18 (25%)	31 (25.6%)	*p* > 0.999	19 (32.2%)	28 (25.4%)	*p* > 0.999
RCA	22 (30.6%)	39 (32.2%)	*p* > 0.999	18 (30.5%)	38 (34.5%)	*p* > 0.999
IM	1 (1.4%)	2 (1.7%)	*p* > 0.999	1 (1.6%)	0	*p* = 0.675
Predilatation balloon:						
(i) Mean diameter (mm)	3.20 ± 0.24	3.24 ± 0.27	*p* = 0.273	3.08 ± 0.31	3.13 ± 0.28	*p* = 0.304
(ii) Mean pressure (atm)	17.75 ± 0.75	17.57 ± 0.91	*p* = 0.209	15.93 ± 0.61	15.82 ± 0.66	*p* = 0.251
Average scaffold number	1.03 ± 0.17	1.07 ± 0.26	*p* = 0.179	1.18 ± 0.39	1.21 ± 0.43	*p* = 0.224
Average scaffold diameter:	3.26 ± 0.25	3.29 ± 0.25	*p* = 0.568	3.22 ± 0.29	3.25 ± 0.31	*p* = 0.345
Average scaffold length (mm)	21.11 ± 3.27	20.62 ± 3.26	*p* = 0.308	22.34 ± 6.87	23.38 ± 7.48	*p* = 0.129
Postdilatation balloon:						
(i) Mean diameter (mm)	3.51 ± 0.31	3.55 ± 0.29	*p* = 0.495	3.32 ± 0.35	3.35 ± 0.35	*p* = 0.535
Mean pressure (atm)	17.69 ± 0.80	17.72 ± 0.83	*p* = 0.924	16.61 ± 0.93	16.76 ± 1.08	*p* = 0.335
(i) 0.0 mm greater than scaffold	12 (16.6%)	19 (15.7%)	*p* = 0.843	42 (71.1%)	78 (70.9%)	*p* = 0.861
(ii) 0.25 mm greater than scaffold	47 (65.2%)	83 (68.6%)	*p* = 0.638	11 (18.6%)	24 (21.8%)	*p* = 0.547
(iii) 0.5 mm greater than scaffold	13 (18.2%)	19(15.7%)	*p* = 0.692	6 (10.1%)	8 (7.2%)	*p* = 0.396
Contrast volume (mL)	153.22 ± 76.76	150.21 ± 57.64	*p* = 0.337	152.49 ± 75.13	146.41 ± 64.91	*p* = 0.625
Radiation dose (mGy)	1120.18 ± 843.89	1014.70 ± 591.75	*p* = 0.934	1396.56 ± 802.95	1162.52 ± 728.34	*p* = 0.029
OCT-guided PCI	13 (18%)	28 (23.1%)	*p* = 0.469	9 (15.2%)	19 (17.2%)	*p* > 0.999
Perforation of vessel	0 (0%)	0	—	0	0	—
Side branch occlusion	0 (0%)	2 (1.6%)	*p* = 0.530	1 (1.6%)	0	*p* = 0.675
Drugs: ASA	72 (100%)	121 (100%)	—	59 (100%)	109 (99.1%)	*p* > 0.999−
Clopidogrel	26 (36.1%)	50 (41.3%)	*p* = 0.543	52 (88.1%)	97 (88.1%)	*p* > 0.999
Ticagrelor	46 (63.9%)	71 (58.7%)	*p* = 0.543	7 (11.8%)	13 (11.8%)	*p* > 0.999
Statin	71 (98.6%)	119 (98.3%)	*p* > 0.999	58 (98.3%)	110 (100%)	*p* > 0.999
ACEI/ARB	61 (84.7%)	100 (82.6%)	*p* > 0.999	55 (93.2%)	99 (90%)	*p* = 0.678
B-blocker	64 (88.8%)	106 (87.6%)	*p* > 0.999	55 (93.2%)	97 (88.1%)	*p* = 0.422

Abbreviations: OCT: optical coherence tomography; PCI: percutaneous coronary intervention; ASA: acetylsalicylic acid; ACEI: angiotensin-converting enzyme inhibitors; ARB: angiotensin receptor blockers.

**Table 3:** Clinical outcomes in both study arms.

	Magmaris group			Ultimaster group		
Clinical outcomes	DM (*N* = 72)	Non-DM (*N* = 121)	*p* value	DM (*N* = 59)	Non-DM (*N* = 110)	*p* value
30-day follow-up						
Primary outcome: cardiac death, myocardial infarction, and stent thrombosis	0 (0%)	0 (0%)	—	2 (3.4%)	0 (0%)	*p* = 0.121
Principal secondary outcome: target lesion failure (cardiac death, target vessel myocardial infract, and target lesion revascularization)	0 (0%)	0 (0%)	—	0 (0%)	0 (0%)	—
Death						
(i) Cardiac	0 (0%)	0 (0%)	—	0 (0%)	0 (0%)	—
(ii) Any	0 (0%)	0 (0%)	—	0 (0%)	0 (0%)	—
Myocardial infarction:						
(i) Target vessel	0 (0%)	0 (0%)	—	0 (0%)	0 (0%)	—
(ii) Any	0 (0%)	0 (0%)	—	2 (3.4%)	0 (0%)	*p* = 0.121
Scaffold:						
(i) Thrombosis	0 (0%)	0 (0%)	—	0 (0%)	0 (0%)	*—*
(ii) Restenosis	0 (0%)	0 (0%)	—	0	0	—
Revascularization:						
(i) Target lesion	0 (0%)	0 (0%)	—	0 (0%)	0 (0%)	*—*
(ii) Target vessel	0 (0%)	0 (0%)	—	0 (0%)	0 (0%)	*—*
(iii) Any	0 (0%)	0 (0%)	—	5 (8.5%)	4 (3.6%)	*p* = 0.279
1-year follow-up						
Primary outcome: cardiac death, myocardial infarction, and stent thrombosis	2 (2.7%)	1 (0.8%)	*p* = 0.557	3 (5.1%)	6 (5.45%)	*p* > 0.999
Principal secondary outcome: target lesion failure (cardiac death, target vessel myocardial infract, and target lesion revascularization)	3 (4.1%)	0 (0%)	*p* = 0.051	2 (3.3%)	4 (3.6%)	*p* > 0.999
Death						
(i) Cardiac	0(0%)	0 (0%)	—	0 (0%)	0 (0%)	*—*
(ii) Any	2 (2.7%)	1 (2.33%)	*p* = 0.138	0 (0%)	0 (0%)	*—*
Myocardial infarction:						
(i) Target vessel	2 (2.7%)	0	*p* = 0.557	1 (1.6%)	4 (3.6%)	*p* = 0.612
(ii) Any	2 (2.7%)	1 (2.33%)	*p* = 0.138	2 (3.4%)	2 (1.8%)	*p* = 0.659
Scaffold:						
(i) Thrombosis	0 (0%)	0	—	0 (0%)	0 (0%)	*—*
(ii) restenosis	2 (2.7%)	0	*p* = 0.138	1 (1.7%)	1 (0.9%)	*p* > 0.999
Revascularization:						
(i) Target lesion	2 (2.7%)	0 (0%)	*p* = 0.138	1 (1.7%)	2 (1.8%)	*p* > 0.999
(ii) Target vessel	3 (2.7%)	0 (0%)	*p* = 0.051	2 (3.4%)	5 (4.5%)	*p* > 0.999
(iii) Any	10 (13.8%)	8 (6.6%)	*p* = 0.124	10 (16.9%)	14 (12.7%)	*p* = 0.492

**Table 4:** Differences in clinical outcomes between the Magmaris and Ultimaster diabetic groups.

Clinical outcomes	Magmaris DM (*N* = 72)	Ultimster DM (*N* = 59)	*p* value
30-day follow-up			
Primary outcome: cardiac death, myocardial infarction, and stent thrombosis	0 (0%)	2 (3.4%)	*p* = 0.201
Principal secondary outcome: target lesion failure (cardiac death, target vessel myocardial infract, and target lesion revascularization)	0 (0%)	0 (0%)	*—*
Death			
(i) Cardiac	0 (0%)	0 (0%)	—
(ii) Any	0 (0%)	0 (0%)	—
Myocardial infarction:			
(i) Target vessel	0 (0%)	0 (0%)	*—*
(ii) Any	0 (0%)	2 (3.4%)	*p* = 0.201
Scaffold: (i) Thrombosis	0 (0%)	0 (0%)	*—*
(ii) restenosis	0 (0%)	0	—
Revascularization:			
(i) Target lesion	0 (0%)	0 (0%)	*—*
(ii) Target vessel	0 (0%)	0 (0%)	*—*
(ii) Any	0 (0%)	5 (8.5%)	*p* = 0.016
1-year follow-up			
Primary outcome: cardiac death, myocardial infarction, and stent thrombosis	2 (2.7%)	3 (5.1%)	*p* = 0.657
Principal secondary outcome: target lesion failure (cardiac death, target vessel myocardial infract, and target lesion revascularization)	3 (4.1%)	2 (3.3%)	*p* > 0.999
Death			
(i) Cardiac	0(0%)	0 (0%)	—
(ii) Any	2 (2.7%)	0 (0%)	*p* = 0.501
Myocardial infarction:			
(i) Target vessel	2 (2.7%)	1 (1.7%)	*p* > 0.999
(ii) Any	2 (2.7%)	2 (3.4%)	*p* > 0.999
Scaffold:			
(i) Thrombosis	0 (0%)	0 (0%)	*p* > 0.999
(ii) Restenosis	2 (2.7%)	1 (1.7%)	*p* > 0.999
Revascularization:			
(i) Target lesion	2 (2.7%)	1 (1.7%)	*p* > 0.999
(ii) Target vessel	3 (2.7%)	2 (3.4%)	*p* > 0.999
(iii) Any	10 (13.8%)	10 (16.9%)	*p* = 0.635

## Data Availability

Data not included in manuscript available on request from corresponding author due to local law and privacy restrictions.
